# RNA sequencing reveals distinct mechanisms underlying BET inhibitor JQ1-mediated modulation of the LPS-induced activation of BV-2 microglial cells

**DOI:** 10.1186/s12974-015-0260-5

**Published:** 2015-02-24

**Authors:** Kyoung Hwa Jung, Amitabh Das, Jin Choul Chai, Sun Hwa Kim, Nishi Morya, Kyoung Sun Park, Young Seek Lee, Young Gyu Chai

**Affiliations:** Department of Molecular and Life Science, Hanyang University, 1271 Sa 3-dong, Ansan, Gyeonggi-do 426-791 South Korea; Department of Bionanotechnology, Hanyang University, 222 Wangsimni-ro, Seoul, 133-791 South Korea

**Keywords:** Anti-inflammatory agents, JQ1, Lipopolysaccharide, Microglia, RNA sequencing

## Abstract

**Background:**

Microglial cells become rapidly activated through interaction with pathogens, and their persistent activation is associated with the production and secretion of various pro-inflammatory genes, cytokines, and chemokines, which may initiate or amplify neurodegenerative diseases. Bromodomain and extraterminal domain (BET) proteins are a group of epigenetic regulators that associate with acetylated histones and facilitate the transcription of target genes. A novel synthetic BET inhibitor, JQ1, was proven to exert immunosuppressive activities by inhibiting the expression of *IL-6* and *Tnf-α* in macrophages. However, a genome-wide search for JQ1 molecular targets is largely unexplored in microglia.

**Methods:**

The present study was aimed at evaluating the anti-inflammatory function and underlying genes targeted by JQ1 in lipopolysaccharide (LPS)-stimulated BV-2 microglial cells using two transcriptomic techniques: global transcriptomic biological duplicate RNA sequencing and quantitative real-time PCR. Associated biological pathways and functional gene ontology were also evaluated.

**Results:**

With a cutoff value of *P* ≤ 0.01 and fold change ≥1.5 log_2_, the expression level of 214 and 301 genes, including pro-inflammatory cytokine, chemokine, and transcription factors, was found to be upregulated in BV-2 cells stimulated with LPS for 2 and 4 h, respectively. Among these annotated genes, we found that JQ1 selectively reduced the expression of 78 and 118 genes (*P* ≤ 0.01, and fold change ≥ 1.5, respectively). Importantly, these inflammatory genes were not affected by JQ1 treatment alone. Furthermore, we confirmed that JQ1 reduced the expression of key inflammation- and immunity-related genes as well as cytokines/chemokines in the supernatants of LPS-treated primary microglial cells isolated from 3-day-old ICR mice. Utilizing functional group analysis, the genes affected by JQ1 were classified into four categories related to biological regulation, immune system processes, and response to stimuli. Moreover, the biological pathways and functional genomics obtained in this study may facilitate the suppression of different key inflammatory genes through JQ1-treated BV-2 microglial cells.

**Conclusions:**

These unprecedented results suggest the BET inhibitor JQ1 as a candidate for the prevention or therapeutic treatment of inflammation-mediated neurodegenerative diseases.

**Electronic supplementary material:**

The online version of this article (doi:10.1186/s12974-015-0260-5) contains supplementary material, which is available to authorized users.

## Introduction

Microglia, a type of glial cell, are resident macrophages of the brain and spinal cord, acting as primary effector cells and regularly participating in host defense and immune surveillance in the brain. These cells play an important role in the brain’s innate immunity and neuronal homeostasis as well as in neuroinflammatory pathologies [1]. Microglial cells become rapidly activated in response to infection, inflammation, or injury, and their activation is associated with the production and secretion of a variety of compounds such as cytotoxic molecules, including reactive oxygen species (ROS), nitric oxide (NO) and prostaglandin E2 (PGE2), and a variety of proinflammatory cytokines, including interleukin *Il-1β*, *Il-6*, and tumor necrosis factor alpha (*Tnf-α*) [2]. Although microglial activation is considered a protective mechanism involved in the clearance of pathogen infection and in regulating tissue repair and recovery, excessive or persistent activation as an uncontrolled immune response stimulates and increases the production of neurotoxic pro-inflammatory mediators and causes neuroinflammation as well as neuronal injury [3]. It has been widely demonstrated that the pathogenesis and progression of several neurological disorders, including Alzheimer’s disease (AD), Parkinson’s disease (PD), brain ischemia, and multiple sclerosis (MS), are associated with the excessive activation of microglia and neuroinflammation [4]. However, the protective mechanisms and the damaging microglial phenotypes have not been fully elucidated. Considering the significant impact of microglial-mediated innate immunity in the central nervous system (CNS), preventing the harmful effects associated with their chronic activation may offer new therapeutic approaches for the treatment of brain injury and neurodegenerative diseases [5].

Microglia expresses a group of pattern recognition receptors (PRRs) to detect and respond to the presence of various stimuli/toxins. Among these, lipopolysaccharide (LPS), the Toll-like receptor 4 (TLR4) ligand, is one of the most potent stimuli for microglial activation. LPS activates intracellular signaling pathways, leading to the secretion of cytokines and to the overexpression of several markers of the immune response. Previous studies have demonstrated that LPS stimulation induces the gene expression of *Tnf-α*, *Il-1β*, *Il-6*, inducible nitric oxide synthase (*iNOS*), and prostaglandin-endoperoxide synthase 2 (*Ptgs-2*) as well as the production of NO and PGE2 in primary and BV-2 microglial cell cultures [6,7]. LPS can reprogram transcription through its ability to activate acetylation of the lysine residues present in histone tails, a general hallmark of gene activation [8]. These acetylated lysines are recognized by highly conserved chromatin readers designated as *N*-terminal bromodomains. These domains are common in all four members of the bromodomain and extraterminal domain (BET) family of adaptor proteins (Brd2, Brd3, Brd4, and Brdt). In humans, at least 40 bromodomain proteins are present, including histone acetyltransferases, helicases, scaffolding proteins, and other cofactors that control gene transcription [9]. These events raise the possibility that bromodomain proteins regulate acetylated, histone-packaged inflammatory gene expression programs associated with various human diseases.

Recently, a potent and highly specific inhibitor, JQ1, of the BET family was discovered by James Bradner and colleagues [9]. This inhibitor competitively binds to BET bromodomain and displaces BET proteins from acetylated lysines on chromatin [9]. They repress downstream gene expression by competitively binding to BET proteins and displacing BET proteins from acetylated lysines on chromatin. These proteins emerged as attractive therapeutic targets in the treatment of inflammation and cancer [9,10]. JQ1 has been shown to control the expression of numerous genes involved in the cell cycle, cell growth, inflammation, and cancer, which suggests that the products of these genes function as epigenetic signaling proteins that regulate transcription in a cell context-dependent manner [7,11,12]. These outcomes promote the possibility of using JQ1 as a potential therapeutic target for modulating gene expression programs associated with a diverse range of pathologies, predominantly cancer and inflammatory diseases. These compounds have been demonstrated to exhibit a potent inhibitory activity against a range of cell lines derived from hematological malignancies, including multiple myeloma, acute myeloid leukemia, Burkitt’s lymphoma, and mixed-lineage leukemia (MLL) [9,12-14]. However, the targeting of BET protein functions by JQ1 in nonmalignant cells remains largely unexplored. Indeed, considering the significance of BET proteins in inflammation, it is important to evaluate the possibility that JQ1 may be exploited as a next-generation anti-inflammatory treatment.

Although JQ1 or I-BET reduces inflammatory gene production in LPS-stimulated macrophages [7,10,11], a genome-wide search for JQ1 molecular targets in LPS-activated BV-2 microglial cells has not yet been performed. We, therefore, performed gene array and comparative gene expression profiling analyses of BV-2 cells treated with LPS, JQ1, or LPS + JQ1 using the precise technique RNA sequencing (RNA-Seq), which is increasingly being used to study gene expression, as it provides unbiased profiles and ability to identify novel transcribed regions compared to microarrays and can be extremely accurate if a sufficient level of coverage is obtained [15,16]. Validation techniques, such as quantitative real-time PCR (qRT-PCR) [17], have corroborated the accuracy of RNA-Seq. To the best of our knowledge, this is the first study to apply these approaches to assess the JQ1-mediated changes in global gene expression in BV-2 microglial cells using RNA-Seq analysis.

Our results show that JQ1 is a potent modulator of microglial activation. In particular, JQ1 treatment resulted in the significant downregulation of key inflammatory genes in LPS-activated BV-2 microglial cells. Importantly, these inflammatory genes were not affected by JQ1 treatment alone. Overall, the results suggested that JQ1 might be an effective therapeutic target with possible research and clinical value. Taken together, these findings establish a role for BET proteins in mouse microglia stimulation and justify the further testing of BET protein-targeting genes in neuroinflammatory diseases.

## Materials and methods

### Cell culture and stimulation

Mouse microglia BV-2 cells were grown in high-glucose Dulbecco’s Modified Eagle’s Medium (DMEM) supplemented with 10% fetal bovine serum (FBS) (catalog # 26140), 100 IU/ml penicillin and 10 μg/ml streptomycin (catalog # 15140) from Invitrogen (Carlsbad, CA, USA). The cells were maintained in a humidified incubator with a 95% air/5% CO_2_ atmosphere at 37°C. JQ1 (+) and JQ1 (−) were purchased from Cayman Chemicals (Ann Arbor, MI, USA) and dissolved in dimethyl sulfoxide (DMSO, Sigma-Aldrich, St. Louis, MO, USA) as a 10-mM stock solution; the stock solution was diluted in DMEM for experiments. The final concentration of DMSO in the medium was less than 10 μL/10 mL, which did not show any effect on cell growth. The cells were treated with a well-tolerated concentration, that is, 500 nM, of JQ1 with LPS (10 ng/mL, Sigma-Aldrich, St. Louis, MO, USA) simultaneously and incubated for 2 and 4 h under normal culture conditions. The medium, with the appropriate agents, was replaced every other day. Primary microglial cells were isolated from 3-day-old ICR mice as previously described [18]. All experimental protocols were conducted in accordance with Institutional Animal Care and Use Committee (IACUC) guidelines and were approved by the IACUC committee at Hanyang University (HY-IACUC-2014-0164A). Briefly, whole brains of neonatal mice were taken; blood vessel and meninges were carefully removed. Then, the whole brains of 12 mice were pooled together, finely minced, and digested with Neural Tissue Dissociation Kit-Postnatal Neurons (Miltenyi Biotec-130-094-802, Auburn, CA, USA). Next, digested cells pass through 70-μm nylon cell strainer (BD Bioscience, San Jose, CA, USA) and were seeded in poly-l-lysine coated T-75 flask in DMEM/nutrient mixture F-12 (DMEM/F12, 1:1) containing 20% FBS (catalog # 26140), 100 IU/ml penicillin and 10 μg/ml streptomycin (catalog # 15140) from Invitrogen (Carlsbad, CA, USA). The cells were maintained in a humidified incubator with a 95% air/5% CO_2_ atmosphere at 37°C. The medium was changed every 2 to 3 days. After 2 weeks in culture, mixed glial cell cultures are shaken at 150 rpm at 37°C for 45 min, and the glial cell suspension was collected from each flask and seeded on poly-l-lysine coated cell culture plate. Microglial cells were sub plated and used for further experiments. More than 96% of cells obtained were microglia as quantified by CD11b (rat monoclonal immunoglobulin G2b (IgG2b), clone M1/70.15.11.5, Miltenyi Biotec Inc., Auburn, CA, USA) FACS analysis (Additional file 1: Figure S1).

### Total RNA extraction

Total RNA (approximately 8 μg) was extracted using TRIzol® (Life Technologies, Carlsbad, CA, USA) according to the manufacturer’s instructions. Briefly, 200 μl of chloroform was added, and the tubes with the lysis mixture were inverted gently for 5 min. The mixture was centrifuged at 12,000 × *g* for 15 min at 4°C, and the clear upper solution was placed into a new tube, to which 500 μl isopropanol was added. The tubes were inverted before incubation on ice for 1 h. The lysis mixture was centrifuged at 12,000 × *g* for 10 min at 4°C, and the isopropanol was decanted. Ice-cold 70% ethanol was added to the RNA pellet for gentle washing. After centrifuging as above for 10 min, the ethanol was removed. The RNA pellets were dried at room temperature for 5 to 10 min before reconstitution in 20 ml RNase-free water, and the RNA was treated with RNase-free DNase (Promega, Madison, WI, USA). The RNA quality was assessed using an Agilent 2100 Bioanalyzer with the RNA 6000 Nano Chip (Agilent Technologies, Waldbronn, Germany), and the quantity was determined using a spectrophotometer (NanoDrop Technologies, Wilmington, DE, USA).

### Quantitative RT-PCR

Reverse transcription of the RNA samples was performed as described [19] using 2 μg of total RNA, 1 μl random hexamers (per reaction), and the Prime Script 1st-strand cDNA synthesis kit (Takara Bio Inc., Shiga, Japan). The random hexamers and RNA templates were mixed and denatured at 65°C for 5 min., followed by cooling for 2 min on ice. Prime Script buffer (5×), RTase and RNAse inhibitor were added to the cooled template mixture and incubated for 1 h at 50°C before enzyme inactivation at 70°C for 15 min. qRT-PCR was performed using SYBR Green PCR Master Mix (Takara Bio Inc., Shiga, Japan) and a 7500 fast real-time PCR system (Applied Biosystems, Foster City, CA, USA). Glyceraldehyde-3-phosphate dehydrogenase (GAPDH) was used as an internal control. Complementary DNA samples were diluted 1.5-fold, and qRT-PCT was performed using an AB-7500 Real-time thermal cycler (Applied Biosystems, Foster City, USA) with SYBR Premix Ex-Taq II (Takara Bio Inc., Shiga, Japan) according to the manufacturer’s directions. The reactions were 20-μl volume with 0.4 mM of each primer (Table 1). Each PCR run included a no-template control with water instead of cDNA and a reverse transcriptase-negative control for each gene. Triplicate measurements were performed for all reactions. Different samples were evaluated using 96-well plates for gene expression experiments, and all samples were analyzed on a single plate for endogenous control determination. The results were analyzed using the critical threshold (∆C_T_) and the comparative critical threshold (∆∆C_T_) methods in the AB-7500 software with the NormFinder and the geNorm PLUS algorithms. The primers were designed using Primer Express (Applied Biosystems, Foster City, USA).

**Table 1:** **List of primers used in qRT-PCR studies**

**Gene designation**	**Forward (5′ → 3′)**	**Reverse (5′ → 3′)**
*Tnf-α*	CAG GCG GTG CCT ATG TCT C	CGA TCA CCC CGA AGT TCA GTA G
*Il1b*	GAA ATG CCA CCT TTT GAC AGT G	CTG GAT GCT CTC ATC AGG ACA
*Cxcl10*	TGC TGG GTC TGA GTG GGA CT	CCC TAT GGC CCT CAT TCT CAC
*Relb*	CCG TAC CTG GTC ATC ACA GAG	CAG TCT CGA AGC TCG ATG GC
*Mcp-1*	TTAAAAACCTGGATCGGAACCAA	GCATTAGCTTCAGATTTACGGGT
*Il-6*	TAGTCCTTCCTACCCCAATTTCC	TTGGTCCTTAGCCACTCCTTC
*Irf-9*	CCTCAGGCAAAGTACGCTG	GGGGTGTCCTATGTCCCCA
*Irak-3*	GTTCTACTCCTGTTCCGTCACC	GTCCCGTTGCTCATATAGGGATA
*Ccl-12*	ATTTCCACACTTCTATGCCTCCT	ATCCAGTATGGTCCTGAAGATCA
*Irf-1*	ATG CCA ATC ACT CGA ATG CG	TTG TAT CGG CCT GTG TGA ATG
*Ccl-7*	CCACATGCTGCTATGTCAAGA	ACACCGACTACTGGTGATCCT
*Ccl-2*	TAA AAA CCT GGA TCG GAA CCA AA	GCA TTA GCT TCA GAT TTA CGG GT
*Ptgs-2*	TTCCAATCCATGTCAAAACCGT	AGTCCGGGTACAGTCACACTT
*Il1a*	TCTATGATGCAAGCTATGGCTCA	CGGCTCTCCTTGAAGGTGA
*Irg-1*	GGCACAGAAGTGTTCCATAAAGT	GAGGCAGGGCTTCCGATAG
*Ccl-4*	TTCCTGCTGTTTCTCTTACACCT	CTGTCTGCCTCTTTTGGTCAG
*Tlr-3*	GTGAGATACAACGTAGCTGACTG	TCCTGCATCCAAGATAGCAAGT
*GAPDH*	TGCGACTTCAACAGCAACTC	CTTGCTCAGTGTCCTTGCTG

### cDNA library preparation for RNA-Seq

Total RNA was extracted from 16 independent samples of BV-2 cells, that is, control 2 h (2 samples), control 4 h (2 samples), JQ1 2 h (2 samples), JQ1 4 h (2 samples), LPS 2 h (2 samples), LPS 4 h (2 samples), LPS + JQ1 2 h (2 samples), and LPS + JQ1 4 h (2 samples) using TRIzol® (Life Technologies, Carlsbad, CA, USA) according to the manufacturer’s protocol. For RNA-Seq, RNA libraries were created from each group using the NEBNext® Ultra™ Directional RNA Library preparation kit from Illumina® (Illumina, San Diego, CA, USA). The first step in the workflow involved the removal of ribosomal RNA using the RNAMius™ Transcriptome Isolation kit (Life Technologies, Carlsbad, CA, USA). Following purification, total RNA was fragmented into small pieces using divalent cations at elevated temperature. The cleaved RNA fragments were copied into first-strand cDNA using reverse transcriptase and random primers, followed by second-strand cDNA synthesis using DNA polymerase I and RNase H. The cDNA fragments were then processed through an end-repair reaction by the addition of a single ‘A’ base, followed by ligation of the adapters. The products of these reactions were then purified and enriched by PCR to create the final cDNA library. The cDNA fragments were sequenced using the Illumina HiSeq2500 (101 cycles PE lane) (National Instrumentation Center for Environmental Management in Seoul National University). Biological replicates (*n* = 2) RNA sequencing was performed on each condition of BV-2 cells: control 2 h (2 samples), control 4 h (2 samples), JQ1 2 h (2 samples), JQ1 4 h (2 samples), LPS 2 h (2 samples), LPS 4 h (2 samples), LPS + JQ1 2 h (2 samples), and LPS + JQ1 4 h (2 samples).

### Differential gene expression analysis

Raw sequence files underwent a quality control analysis using FastQC (version 0.10.1, http://www.bioinformatics.babraham.ac.uk/projects/fastqc/). To avoid low-quality data, we clipped and trimmed the reads using FASTX-Toolkit (version 0.0.14, http://hannonlab.cshl.edu/fastx_toolkit/). For the analysis of differentially expressed genes, the data of quality-checked reads for each condition were processed with the TopHat (version 2.0.10) [20] software based on reference genome sequence (*Mus musculus* University of California, Santa Cruz (UCSC) mm10), and the differential gene expressed values of each sample were calculated by Cufflinks [21] based on ‘fragments per kilobase per million map reads’ (FPKM) methods [22]. The data from each condition were combined separately, to produce eight biological datasets, and the genes whose levels of expression significantly differed were identified. We used a 1% false discovery rate (FDR), *P* ≤ 0.01, and fold change ≥1.5 log_2_ for up- or downregulation as the criteria for defining differentially expressed genes. RNA-Seq experiments were visualized using HOMER (version 4.7) after preparing custom tracks for the UCSC Genome Browser (http://genome.ucsc.edu/). The data acquired were deposited in the Gene Expression Omnibus database under dataset accession numbers SRX683618, SRX683736, SRX683740, SRX683672, SRX683738, SRX683742, SRX683671, SRX683737, SRX683741, SRX683675, SRX683739, and SRX683743.

### Functional annotation and pathways

Database for Annotation, Visualization and Integrated Discovery (DAVID) (version 6.7) software (http://david.abcc.ncifcrf.gov/home.jsp) was used to determine the most functional annotation of significant genes in datasets, as described previously [23]. DAVID calculates a modified Fisher’s exact *P* value to demonstrate gene ontology (GO) or molecular pathway enrichment. Values less than 0.05 were considered to be strongly enriched in the annotation category. To determine the possible biological pathways involved in JQ1-treated BV-2 cells, a gene classification analysis of the downregulated genes was performed using the PANTHER classification system version 9.0 (http://www.pantherdb.org), as described previously [24]. Genes from datasets that were associated with biological pathways in the PANTHER Pathways Knowledge Base were considered for literary analysis.

### Enzyme-linked immunosorbent assay

Primary microglial cells were cultured in the same condition as above. Primary microglial cells were treated with LPS (10 ng/ml), JQ1 (500 nM), and LPS (10 ng/ml) + JQ1 (500 nM) for 2 and 4 h. After treatment, the concentration of the pro-inflammatory mediators Ccl2, Ccl7, and Cxcl10 were determined in cell culture supernatants using the mouse enzyme-linked immunosorbent assay (ELISA) kit (Komabiotec, Seoul, Korea) according to the manufacturer’s protocol.

### Statistical analysis

The data were analyzed using Origin Pro 8 (Origin Lab Corporation, Northampton, MA, USA). Each value is expressed as the mean ± standard error of the mean (SEM). The statistical analysis was performed using SPSS 17.0 (SPSS Inc., Chicago, IL, USA). The data were tested by a one-way ANOVA, followed by Tukey’s HSD *post hoc* test. *P* < 0.05 and *P* < 0.001 were considered significant.

## Results

### Gene-induction patterns in LPS-stimulated BV-2 microglial cells

**Figure 1:**
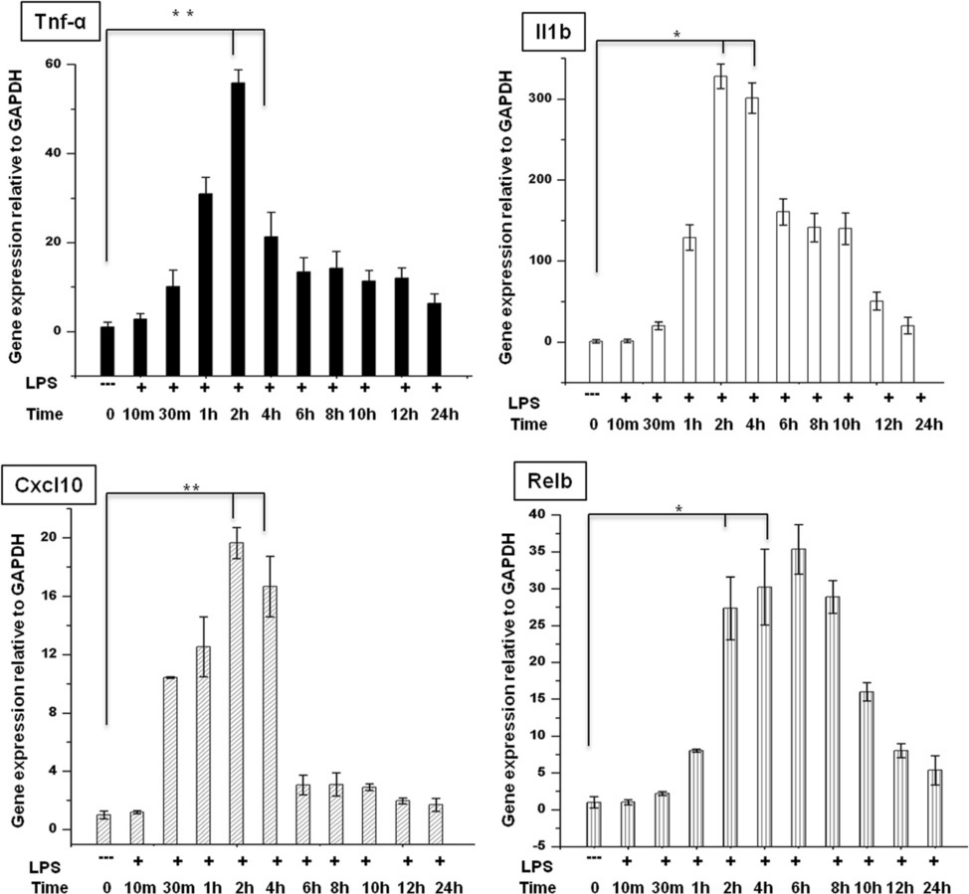
**Induction of inflammatory response-related genes in LPS-stimulated BV-2 microglial cells.** Quantitative real-time reverse transcriptase PCR analysis of the expression of inflammatory genes in BV-2 microglial cells stimulated with LPS (10 ng/mL). Inflammatory genes were significantly upregulated in cells treated with LPS compared to untreated cells (*P < 0.05 and **P < 0.001) at the indicated times. Gene expression was normalized to *GAPDH* transcript levels. The data represent three independent experiments. The values are the mean ± SD of triplicate wells. LPS, lipopolysaccharide; *GAPDH*, glyceraldehyde-3-phosphate dehydrogenase; *Tnf-α*, tumor necrosis factor alpha.

We began our study by examining the timing of gene activation induced by LPS by performing an expression analysis of BV-2 microglial cells treated with LPS (10 ng/mL) for 10 min to 24 h and compared the results with the expression in untreated cells under normal culture conditions. We found a significant, time-dependent upregulation of inflammatory response-related genes with up to 4 h of LPS treatment (Figure 1). Because we found that most of the inflammatory response-related genes were upregulated at the 2- and 4-h time points, we chose of these time points for transcriptional profiling; these time points were also used in other studies [7,25,26] that investigated the general induction pattern of microglial activation by LPS.

### Distinct gene signatures are identified during the inflammatory response according to RNA-Seq analysis

**Figure 2:**
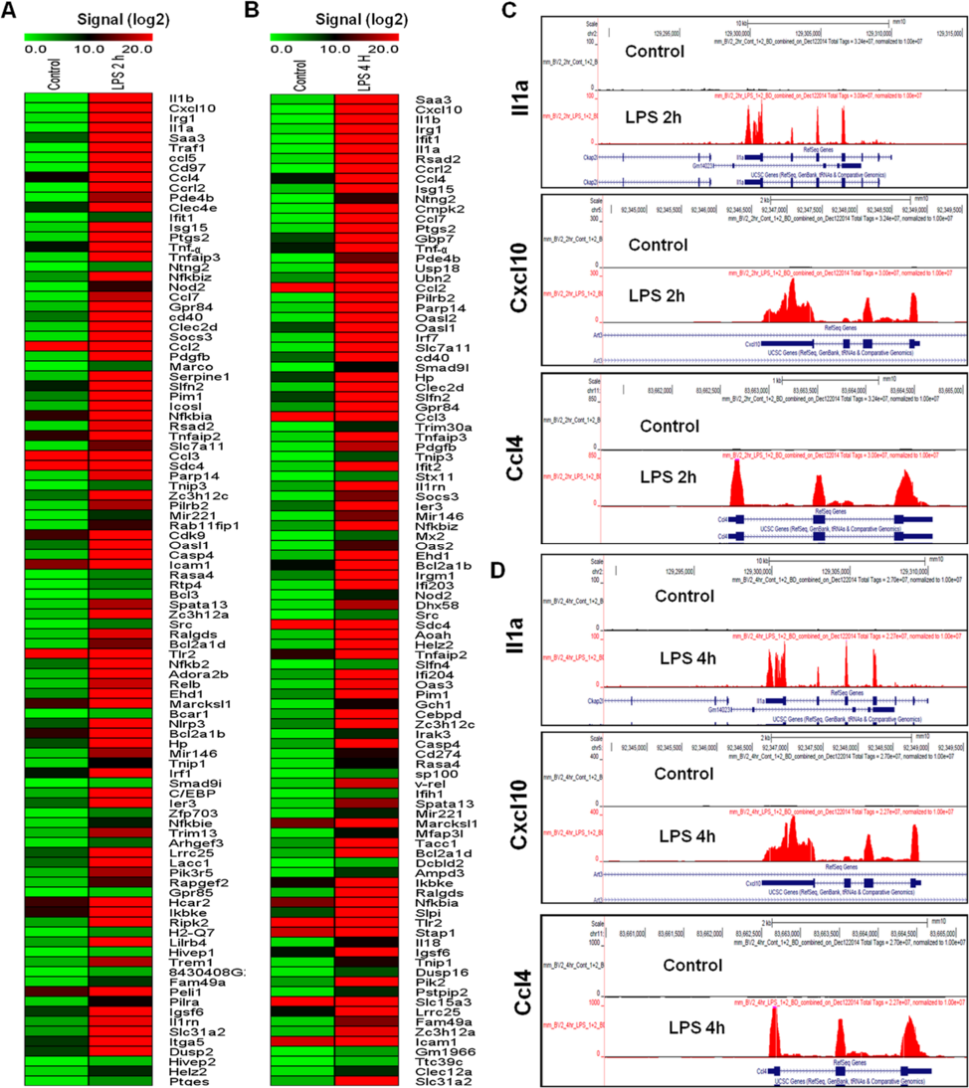
**RNA-Seq analysis reveals LPS-stimulated pro-inflammatory gene expression in BV-2 microglial cells. (A and** **B)** A heat map representing RNA-Seq gene expression of top 100 upregulated (*P* ≤ 0.01 and fold change ≥1.5 log_2_) inflammatory genes in 2- and 4-h LPS-stimulated BV-2 microglial cells compared to the control, respectively. Biological replicates (*n* = 2) for each condition were combined separately, and the heat map were generated with the Multi Experiment Viewer (version 4.8) software. **(C and** **D)** UCSC Browser images representing the normalized RNA-Seq read density in 2- and 4-h LPS-stimulated BV-2 microglial cells compared to the control, respectively. LPS, lipopolysaccharide.

**Figure 3:**
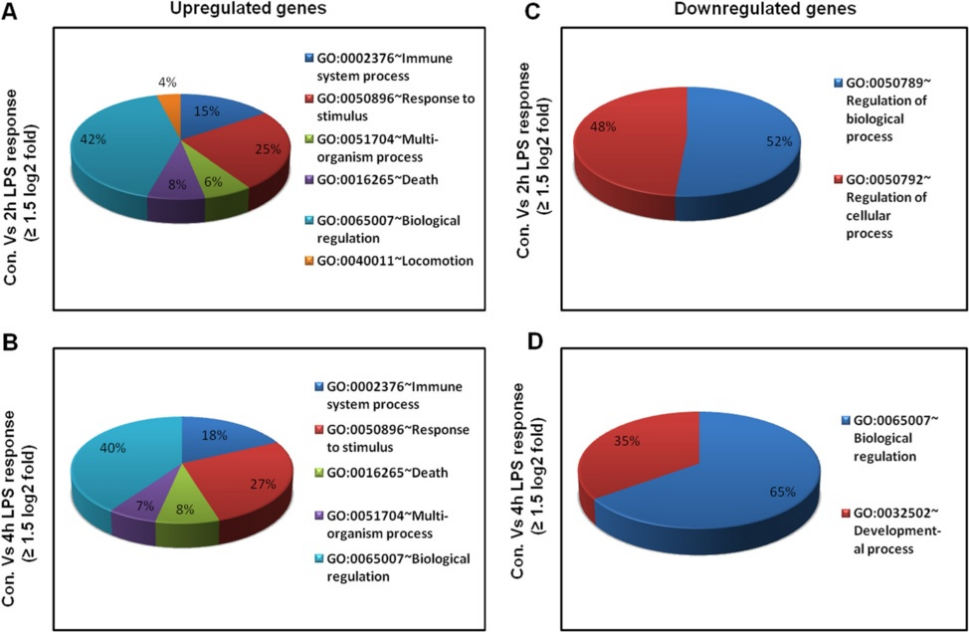
**Functional annotations of LPS-inducible genes. (A and B)** Gene Ontology analysis of functional annotations (biological process) associated with 2- and 4-h LPS-inducible upregulated genes in BV-2 microglia in comparison with the control, respectively. **(C and D)** Gene Ontology analysis of functional annotations (biological process) associated with 2- and 4-h LPS-inducible downregulated genes in BV-2 microglia in comparison with the control, respectively. LPS, lipopolysaccharide; GO, gene ontology.

To identify the response of BV-2 cells to LPS (10 ng/mL), BV-2 cells were stimulated for two different time periods, that is, 2 and 4 h. RNA-Seq analysis revealed differentially expressed genes in the LPS-stimulated BV-2 cells at both time points: 270 genes for 2 h and 396 genes for 4 h (increased and decreased in expression fold change ≥1.5 log_2_ and *P* ≤ 0.01, respectively) were differentially regulated. Among them, 214 and 301 genes were upregulated, whereas 56 and 95 genes were downregulated at 2 and 4 h, respectively, after LPS treatment (Figure 2A,B and Additional file 2: Table S1 and S2). Notably, most of the upregulated genes included the following inflammatory response- and immune response-related genes: *iNOS*, interleukin and interleukin-related genes (*Il1-β*, *Il1a*, *Il18*, *Il1rn*); *Tnf-α* and *Tnf-α*-related genes (*Tnfaip3*, *Tnip3*, *Tnip1*, *Tnfaip2*); a prostaglandin-related gene, *Ptgs2*; *NF-κB*-related genes (*Nfkbiz*, *Nfkbia*, *Nfkb2*, *Relb*, *Nfkbie*, *Nfkb1*); interferon-related genes (*Ifit1*, interferon regulatory factors (*Irf*) *Irf1*, *Irf7*, *Irf9*); and cytokines or chemokines (*Cxcl10*, *Ccl4*, *Ccl7*, *Ccl2*, *Ccl3*, *Ccl12*, *Ccl9*) (Figure 2A,B,C,D). We selected these genes based on their biological processes and the molecular functions of their gene ontology. As the downregulated genes were not associated with inflammation, only upregulated genes were studied further. We confirmed by a GO analysis (FDR 0.05) using DAVID Bioinformatics Resources that LPS downregulated transcripts were associated with regulation of biological and cellular processes in BV-2 microglial cells (Figure 3C,D).

### BET inhibitor JQ1 reduces inflammatory responses in BV-2 microglial cells

**Figure 4:**
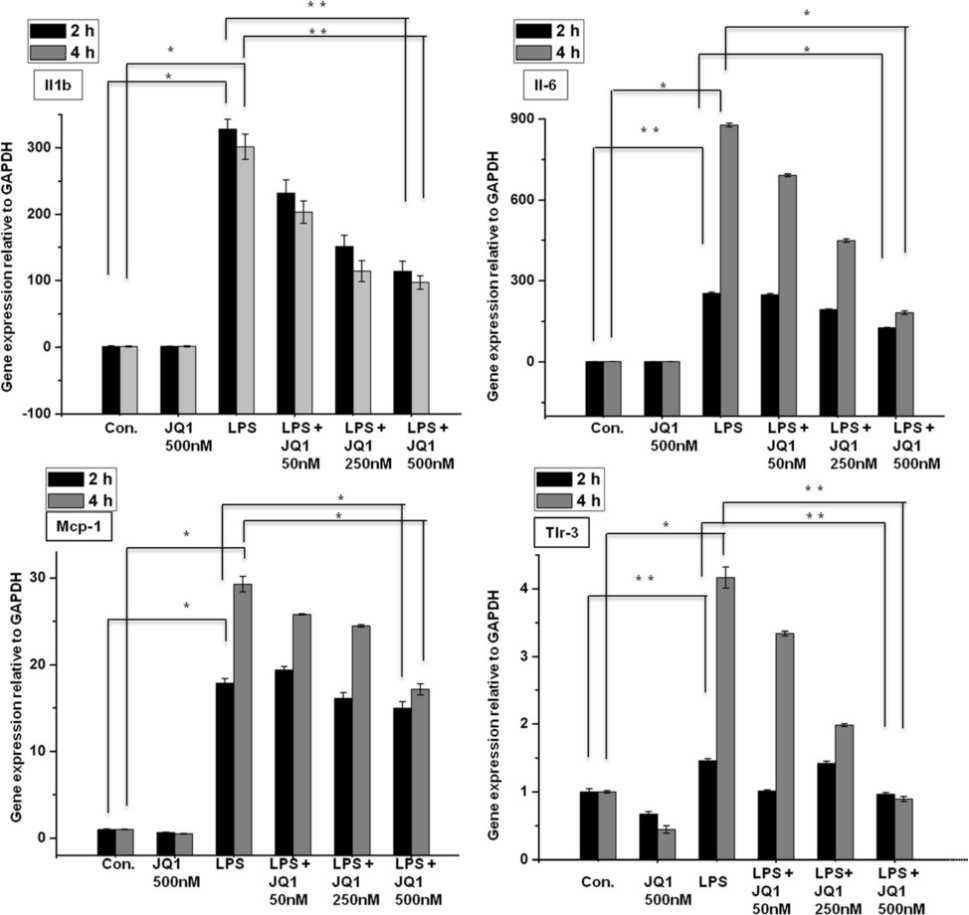
**Inhibitory effect of JQ1 on LPS-induced BV-2 microglial cells.** BV-2 microglial cells were treated with different concentrations of JQ1 for 2 and 4 h, followed by treatment with LPS (10 ng/ml). Inflammatory genes were significantly downregulated in cells treated with JQ1 compared to untreated cells (*P < 0.05 and **P < 0.001) at the indicated times. Gene expression was normalized to GAPDH transcript levels. The data represent three independent experiments. The values are the mean ± SD of triplicate wells. LPS, lipopolysaccharide; *GAPDH*, glyceraldehyde-3-phosphate dehydrogenase; Con., control.

**Figure 5:**
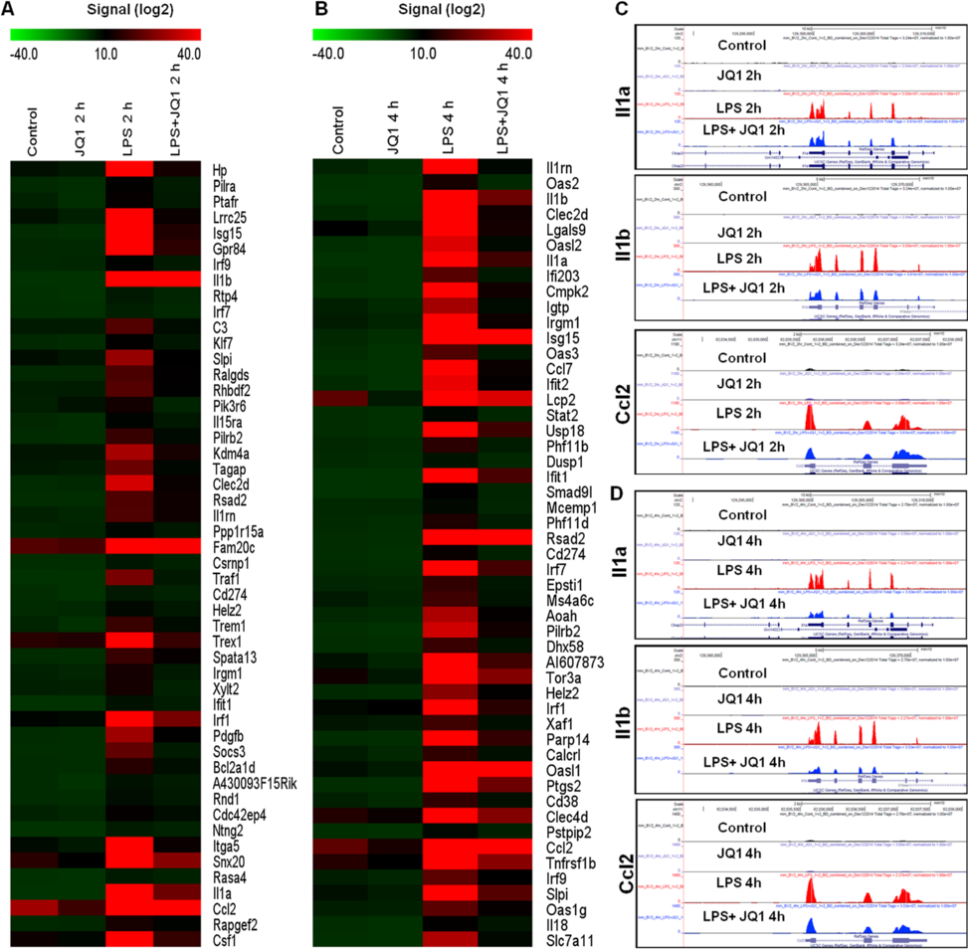
**JQ1 suppresses a specific subset of LPS-inducible genes. (A)** Heat map representation of the top 50 expression levels of genes that were downregulated (*P* ≤ 0.01 and fold change ≥1.5) by JQ1 at 2 h (left panel) and **(B)** 4 h (right panel) after LPS stimulation of two independent BV-2 microglial cultures. Biological replicates (*n* = 2) for each condition were combined separately, and the heat map were generated with the Multi Experiment Viewer (version 4.8) software. **(C and D)** UCSC Browser images representing the normalized RNA-Seq read density in JQ1-downregulated inflammatory genes at 2 and 4 h in LPS-stimulated BV-2 microglial cells compared to the control, respectively. LPS, lipopolysaccharide.

To investigate whether the broad-spectrum BET protein inhibitor JQ1 is also a broad-spectrum, anti-inflammatory agent, we tested its efficacy as an immunomodulatory drug that could counter microglia-mediated inflammation. We first examined whether JQ1 could alter the expression of inflammation-related genes in microglial cells. The effect of JQ1 on LPS-induced inflammatory genes was examined at 2 and 4 h of LPS stimulation. Most of the genes were significantly suppressed by JQ1 in a dose-dependent manner (Figure 4); indeed, we observed that 500-nM JQ1, a dose used in previous publications [9,27,28], led to a marked reduction of inflammatory gene expression in BV-2 microglial cells. Furthermore, we exposed BV-2 microglial cells to LPS and treated them biologically inactive enantiomer JQ1 (−). As expected, expression levels of inflammatory genes were not reduced in JQ1 (−)-treated BV-2 microglial cells (Additional file 3: Figure S2). We then exposed the BV-2 microglial cells to LPS and concomitantly treated them with JQ1 (+) for both time periods and compared the gene expression profile from the group treated with LPS alone with that obtained from the group treated with JQ1 + LPS. Treatment of BV-2 microglial cells with JQ1 and LPS resulted in the downregulation (*P* ≤ 0.01, and fold change ≥1.5) of 78 and 118 of the LPS-inducible genes at 2 and 4 h, respectively (Figure 5). JQ1 suppressed the expression of key LPS-inducible inflammation- and immunity-related genes, including *Il1a*, *Il1b*, *Irg1*, *Ptgs2*, *iNOS*, *Ccl2*, *Ccl4*, *Ccl7*, *Ccl12*, *Cxcl10*, *Irf1*, *Irf7*, and *Irf9* (Figure 5A-D). Most interestingly, an inhibitor of the *NF-κB* transcription factor, *Nfkbia*, was not suppressed by JQ1. A crucial inflammatory gene, *Tnf-α* (Additional file 4: Figure S3), as well as other inflammation- and immunity-related genes, such as *Saa3*, *Nfkbiz*, *Tnfaip2*, *Nfκb2*, and *Ccl3*, were unaffected by JQ1, suggesting that JQ1-treated, LPS-inducible gene expression is highly selective. Consistent with our findings, Nicodeme *et al*. [7] reported that significant inflammatory genes *Tnf-α*, *Ccl3* were unaffected by another synthetic BET family proteins (I-BET) in bone marrow-derived macrophages (BMDM). They observed that following I-BET treatment higher BET levels at *Tnf-α* locus were associated with largely unchanged levels of positive transcriptional elongation factor b, RNA polymerase II, and RNA polymerase II S2. In contrast, Belkina *et al*. [10] demonstrated that JQ1 is a potent inhibitor of *Tnf-α* production in BMDM. This mechanism is the subject of ongoing investigations. This is an exciting area that we are keenly pursuing further.

### Effect of JQ1 alone on resting BV-2 microglial cells

We also evaluated the effect of JQ1 alone in resting BV-2 cells. The results showed that JQ1 alone, in the absence of LPS stimulation, also altered the expression of some genes, with a 1.5 log_2_-fold cutoff value. Most of these genes have no well-established role in CNS inflammation, whereas some genes (*Ptgs2*, *iNOS*, *Il1-β*, *Il1a*, *Il18*, *Il1rn*, *Tnf-α*, *Tnfaip3*, *Tnip3*, *Tnip1*, *Tnfaip2*, *Ifit1*, *Irf1*, *Irf7*, *Irf9*, *Cxcl10*, *Ccl4*, *Ccl7*, *Ccl2*, *Ccl3*, *Ccl12*, *Ccl9*) associated with inflammation were expressed marginally or insignificantly. A total 67 genes (*P* ≤ 0.01 and fold change ≥1.5) for 2 h and 64 genes (*P* ≤ 0.01 and fold change ≥1.5) for 4 h were upregulated in the BV-2 microglial cells treated with JQ1 alone. Notably, we observed that the DDHD domain containing 1 ghrelin, small nucleolar RNA, C/D box 42A for 2 h and Rab geranylgeranyl transferase, b subunit, eukaryotic translation initiation factor 4, gamma 1, small nucleolar RNA, and H/ACA box 41 genes for 4 h were upregulated in JQ1 stimulated BV-2 microglial cells (Additional file 5: Table S3 and S4). Based on the literature review, these genes have no well-established role in CNS inflammation. In addition, we confirmed by a GO analysis (FDR 0.05) using DAVID Bioinformatics Resources that JQ1 upregulated transcripts associated with cellular macromolecular complex assembly and primary metabolic processes (Additional file 6: Figure S4). Interestingly, Banerjee *et al*. [29] reported that JQ1 showed potent upregulation of chromatin modification genes, including *Sirt1*, *Hdac6*, and multiple lysine demethylases (KDMs) as well as *Hexim-1* in the J-Lat 10.6 cells, which have potential role for HIV reactivation. In our RNA-Seq data, we could not identify any chromatin modification genes induced by JQ1. However, we observed JQ1 slightly upregulated *Hexim-1* in BV-2 microglial cells. Nevertheless, whether these genes have any functional role on JQ1-mediated modulation of microglia activation will require further study.

### Functional and pathways analyses of JQ1 in LPS-stimulated BV-2 microglial cells

**Figure 6:**
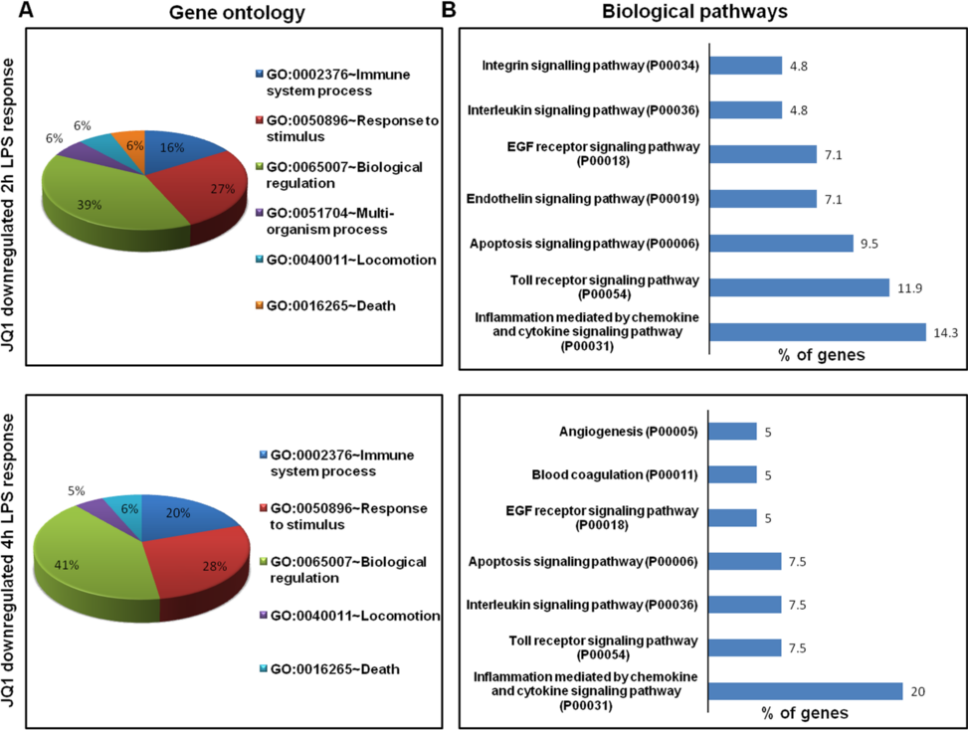
**Functional annotation and biological pathways of the JQ1-downregulated genes. (A)** Analysis of GO term enrichment for the ‘biological process’ category of JQ1 downregulated genes. The top GO terms are ranked by the number of counts. **(B)** The most highly represented biological pathways of JQ1 downregulated genes in BV-2 microglial cells. GO, gene ontology.

The groups of LPS upregulated genes that showed a change in expression of (*P* ≤ 0.01 and fold change ≥1.5 log_2_) were subjected to a GO analysis (FDR 0.05), with functional annotation using DAVID Bioinformatics Resources and KEGG (Kyoto Encyclopedia of Genes and Genomes) pathways. DAVID revealed that all major biological processes and molecular functions within GO for the LPS upregulated transcripts were, for the most part, genes associated with the immune system process, response to stimulus, and biological regulation (Figure 3A,B). To further functionally classify the JQ1 downregulated genes (*P* ≤ 0.01 and fold change ≥1.5) with LPS stimulation, we again used DAVID Bioinformatics Resources. Interestingly, we observed that the largest groups of genes are involved in the same biological processes, that is, the immune system process, response to stimulus, and biological regulation (Figure 6A). To determine the possible biological pathways of the JQ1 downregulated genes (*P* ≤ 0.01 and fold change ≥1.5) in LPS-treated BV-2 cells, we utilized PANTHER classification system version 9.0. The major categories of the biological pathways were inflammation mediated by chemokine and cytokine, Toll receptor, and interleukin signaling pathways (Figure 6B).

### Confirmation of differentially expressed genes by qRT-PCR

**Figure 7:**
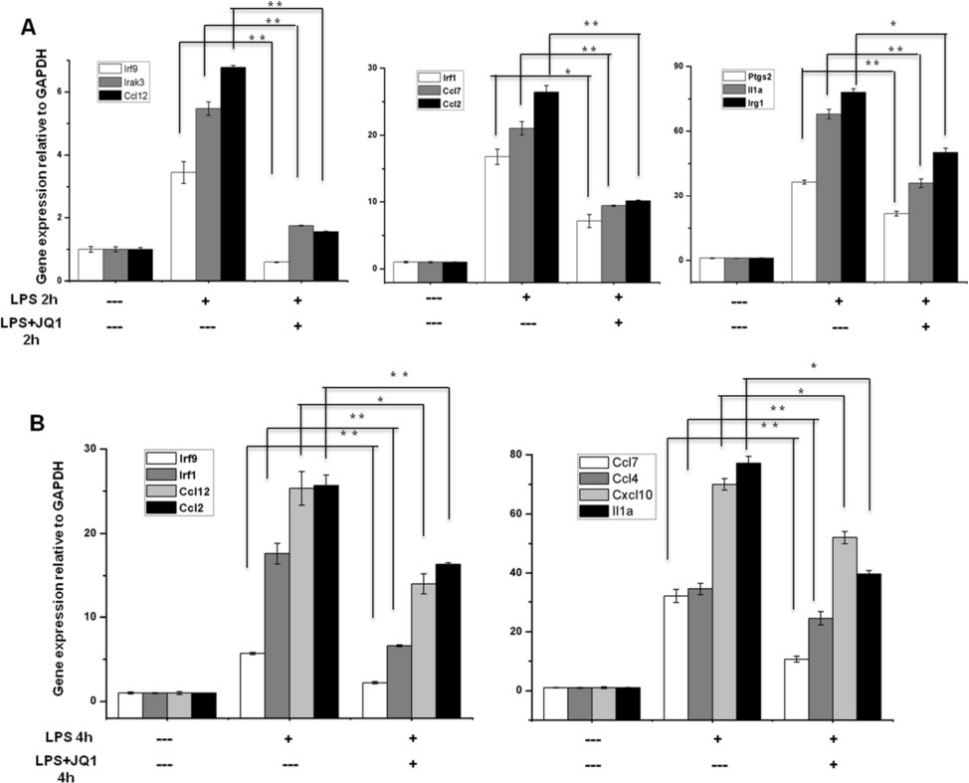
**Confirmation of differentially expressed genes by quantitative reverse transcription-polymerase chain reaction. (A** and **B)** The *Irf9*, *Irf1*, *Irak3*, *Ccl2*, *Ccl7*, *Ccl4*, *Ccl12*, *Cxcl10*, *Ptgs2*, *Irg1*, and *Il1a* genes were significantly downregulated in JQ1-treated BV-2 microglial cells. Gene expression was normalized to GAPDH transcript levels. *P < 0.05 and **P < 0.001 compared with the control. The data represent three independent experiments. LPS, lipopolysaccharide; GAPDH, glyceraldehyde-3-phosphate dehydrogenase.

**Figure 8:**
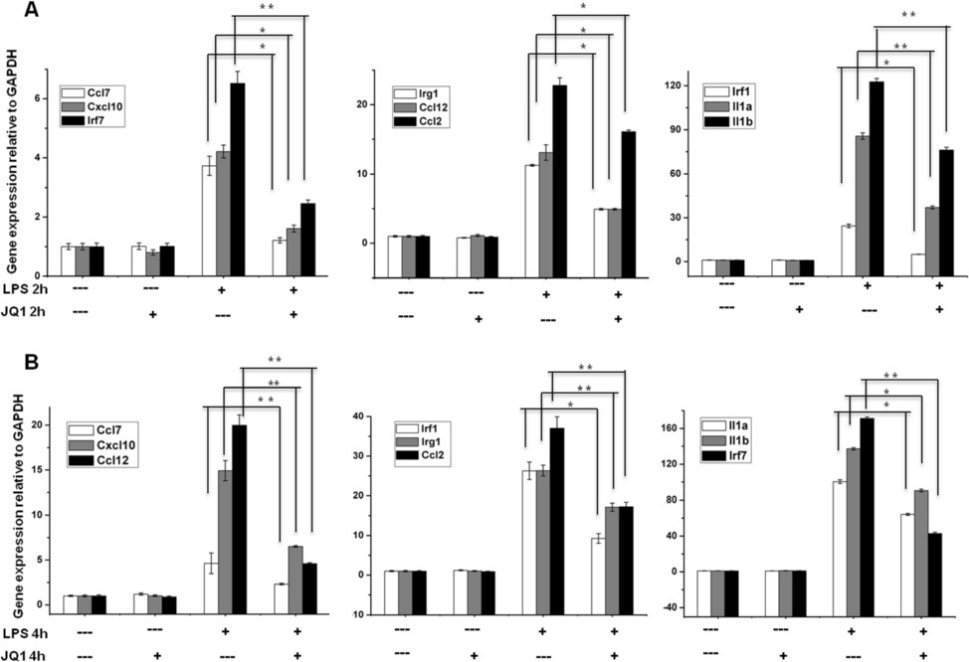
**The BET family bromodomain inhibitor JQ1 reduces LPS induced pro-inflammatory genes in primary microglial cells. (A** and **B)** The *Ccl7*, *Cxcl10*, *Irf7*, *Irg1*, *Ccl12*, *Ccl2*, *Irf1*, *Il1a*, and *Il1b* genes were significantly downregulated in JQ1 (500 nM)-treated primary microglial cells at 2 and 4 h under inflammatory conditions (LPS 10 ng/mL). Gene expression was normalized to *GAPDH* transcript levels. *P < 0.05 and **P < 0.001 compared with the control. The data represent three independent experiments. LPS, lipopolysaccharide; *GAPDH*, glyceraldehyde-3-phosphate dehydrogenase.

**Figure 9:**
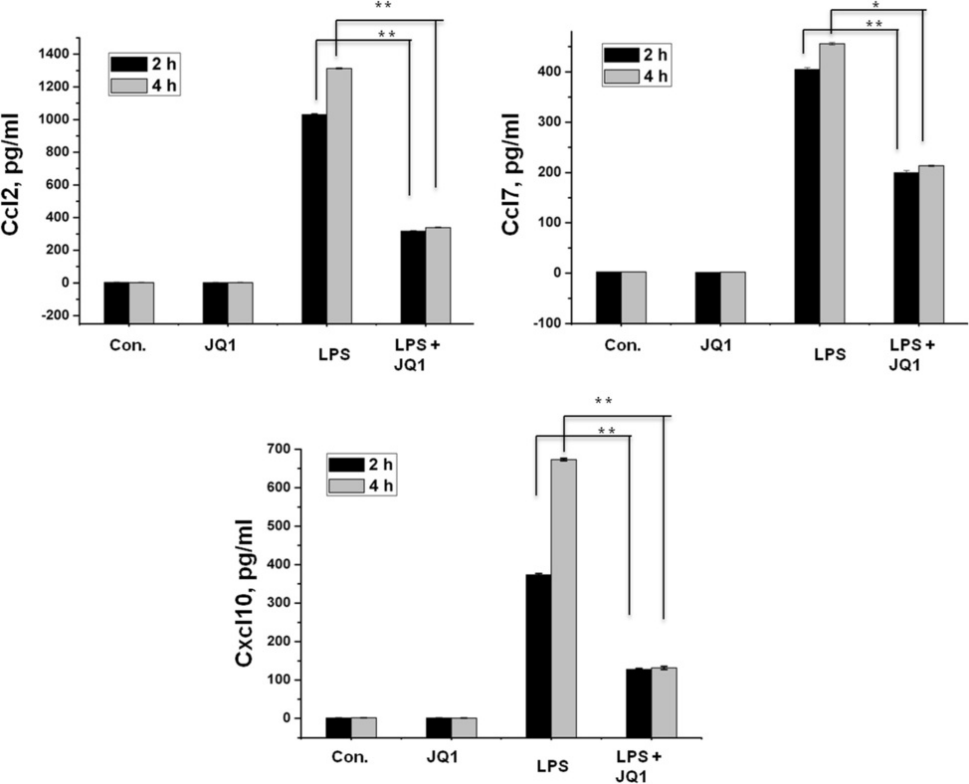
**The BET family bromodomain inhibitor JQ1 reduces LPS-induced release of pro-inflammatory mediators.** Primary microglial cell culture supernatants of LPS and/or JQ1 co-treated cells were subjected to ELISA to detect the levels of pro-inflammatory cytokines/chemokines. Therefore, primary microglial cells were treated with 10 ng/mL LPS and/or 500 nM of JQ1 for 2 and 4 h, followed by quantification of Ccl2, Ccl7, and Cxcl10 levels. Values are given in pg/ml. Means and standard deviations of the mean of the three independent experiments are shown (*P value <0.05, **P value <0.001). LPS, lipopolysaccharide; Con., control.

A large number of genes that were identified as differentially regulated by the RNA-Seq analysis were subjected to validation by qRT-PCR using *GAPDH* as the reference gene. Most were selected to be validated according to the distinct effects of JQ1 on the LPS-affected genes. To measure gene expression, mRNA was reverse transcribed into cDNA using Prime Script TM Reverse Transcriptase (Takara Bio Inc., Shiga, Japan); the qRT-PCR assays were repeated several times using at least three mRNA preparations from independent experiments. The results are expressed as the fold change relative to control levels. Thirteen genes were selected for verification; the RNA-Seq expression pattern confirmed for eleven (*Irf9*, *Irf1*, *Irak3*, *Ccl2*, *Ccl7*, *Ccl4*, *Ccl12*, *Cxcl10*, *Ptgs2*, *Irg1*, *Il1a*; Figure 7A,B), and two were non-significant (data not shown) in the qRT-PCR analysis compared to the RNA-Seq experiments. Overall, the qRT-PCR data correlated with the RNA-Seq data (Tables 2 and 3). To confirm the distinct effects of JQ1 in primary microglial, we incubated primary microglial cells under inflammatory conditions (LPS 10 ng/mL), which induced inflammatory genes. More importantly, JQ1 suppressed the expression of key LPS-inducible inflammation- and immunity-related genes, including *Ccl7*, *Cxcl10*, *Irf7*, *Irg1*, *Ccl12*, *Ccl2*, *Irf1*, *Il1a* and *Il1b*, in primary microglial cells (Figure 8A,B). However, it should be noted that *Ptgs2* gene was not affected by the treatment of LPS. In addition, we analyzed cytokines/chemokines in the supernatants of treated primary microglial cells with ELISAs. Compared to untreated cells Ccl2, Ccl7, and Cxcl10 in the supernatants were increased in primary microglial cells following 2 and 4 h LPS (10 ng/mL) treatment. Co-treatment with JQ1 (500 nM) led to significant reduction of Ccl2, Ccl7, and Cxcl10 in primary microglial cells (Figure 9).

**Table 2:** **Comparison of RNA-Seq and qRT-PCR data in 2 h JQ1 and LPS-treated BV-2 microglia cells**

**RNA-Seq fold change**				**qRT-PCR fold change**	
**Gene symbol**	**Gene accession ID**	**LPS_2 h**	**LPS + JQ1_2 h**	**LPS_2 h**	**LPS + JQ1_2 h**
*Ccl12*	NM_011331	9.89	2.19	6.78	1.56
*Il1a*	NM_010554	57.04	34.96	67.98	36.02
*Irf9*	NM_001159418	3.23	0.59	3.45	0.59
*Ptgs2*	NM_011198	32.41	25.05	36.5	21.68
*Irak3*	NM_028679	3.44	2.11	5.47	1.75
*Irf1*	NM_001159393	14.31	3.46	16.79	7.15
*Ccl2*	NM_011333	24.67	10.23	26.44	10.16
*Irg1*	NM_008392	56.15	51.20	78.09	50.19
*Ccl7*	NM_013654	17.41	14.23	21.03	9.428

**Table 3:** **Comparison of RNA-Seq and qRT-PCR data in 4 h JQ1 and LPS-treated BV-2 microglia cells**

**RNA-Seq fold change**				**qRT-PCR fold change**	
**Gene symbol**	**Gene accession ID**	**LPS_4 h**	**LPS + JQ1_4 h**	**LPS_4 h**	**LPS + JQ1_4 h**
*Ccl12*	NM_011331	29.79	13.89	25.33	13.99
*Il1a*	NM_010554	66.01	31.18	77.23	39.68
*Ccl7*	NM_013654	39.89	18.24	32.02	10.68
*Irf1*	NM_001159393	17.02	4.25	17.61	6.63
*Irf9*	NM_001159418	4.89	2.90	5.71	2.23
*Cxcl10*	NM_021274	88.25	82.02	70.02	52.03
*Ccl2*	NM_011333	42.15	21.03	25.69	16.35
*Ccl4*	NM_013652	41.02	34.01	34.52	24.55

## Discussion

The BET family comprises a distinct group of epigenetic regulators governing the assembly of histone acetylation-dependent chromatin complexes that regulate inflammatory gene expression [30]. There are several small molecule BET inhibitors targeting diverse BET family members in cancer and inflammatory diseases [31]. For example, a pan-BET inhibitor, I-BET, has been proven to protect against LPS-induced endotoxic shock [7]. Another BET inhibitor can disrupt the T-cell-mediated inflammatory response [32]. Among these inhibitors, JQ1 has attracted the most attention because of its significant efficiency in hematological malignancies [33]. Recently, other studies have reported even wider prospective applications for JQ1, such as in attenuating lung fibrosis [34], endotoxemic shock [10], NO synthesis, and innate immunity [35], suggesting that JQ1 may have anti-inflammatory activity. However, none of these studies addressed the effects of JQ1 at the genome-wide expression level in BV-2 microglial cells. We examined BV-2 cell lines as a model of inflammation studies. This is one of the major uses of microglia. Previously, other reports demonstrated that BV-2 cell lines have close resemblance to primary brain microglia [36-38]. Since BV-2 cells are easy to culture, they are an important tool to study not only inflammatory processes [38] but also phagocytosis [39]. In the present study, we, for the first time, showed the anti-inflammatory effect of JQ1 on genome-wide mRNA levels in BV-2 microglial cells, a model system for studying inflammation, using RNA-Seq analysis. This study provides the most comprehensive analysis thus far, as the technique provides unbiased profiles, ability to identify novel transcribed regions, compared to microarrays, and can be extremely accurate. This unbiased profiling approach revealed that the importance of BET proteins in the regulation of key inflammatory genes involved in the establishment of innate immunity in BV-2 microglial cells.

The results show that the stimulation of BV-2 microglial cells with LPS upregulated numerous inflammatory genes, including *Nos2*, *Il1b*, *Il1a*, *Il18*, *Il1rn*, *Tnf-α*, *Ptgs2*, *Nfκbiz*, *Nfκbia*, *Nfκb2*, *Relb*, *Nfκbie*, *Nfκb1*, *Ifit1*, *Irf1*, *Irf7*, *Irf9*, *Cxcl10*, *Ccl4*, *Ccl7*, *Ccl2*, *Ccl3*, *Ccl12*, and *Ccl9*. Treatment of BV-2 microglial cells with JQ1 resulted in the downregulation of 78 and 118 (*P* ≤ 0.01 and fold change ≥1.5) of the LPS-inducible genes at 2 and 4 h, respectively, suppressing key LPS-inducible inflammatory genes, including *Il1a*, *Il1b*, *Nos2*, *Ptgs2*, *Irf1*, *Irf7*, *Irf9*, *Ccl2*, *Ccl7*, *Ccl9*, *Ccl12* and *Cxcl10* (Figure 5A,B). *Il1* is the most widely studied pro-inflammatory gene; the extensively characterized forms of *Il1* are *Il1a* and *Il1b* [40]. *Il1a* and *Il1b* play a crucial role in the development of AD and PD, the pathogenic hallmark of which is CNS inflammation [41,42]. Following CNS damage, *Il1* is rapidly released from activated microglia, and an elevated level of the *Il1* cytokine is an important hallmark of neuroinflammation [43]. In this study, we showed that JQ1 treatment significantly reduced the expression of *Il1a* and *Il1b*, which had been increased by LPS stimulation. Thus, the downregulation of *Il1a* and *Il1b* through JQ1 could inhibit neuroinflammation as well as neurodegenerative disorders.

RNA-Seq revealed treatment that JQ1 inhibited the expression of important chemokines in LPS-activated microglial cells, for example, *Ccl2*, *Ccl7*, *Ccl12*, and *Cxcl10* (Figure 5A,B). These chemokines, also referred to as inflammatory cytokines, and their excessive production have been associated with disease progression and severe inflammation pathologies, including MS [44]. Conductier *et al*. [45] reported that *Ccl2* plays a crucial role in neuroinflammatory diseases and also considered it as a target in the treatment of neuroinflammatory disorders. *Ccl2* and *Ccl7* are highly expressed during MS in microglia, astrocytes, and other inflammatory cells [46]*. Ccl12* also has an inflammatory role, as its level is upregulated in both microglia and astrocytes when stimulated with the proinflammatory cytokine *Il17* [47]. The expression of CXC chemokine ligand 10, *Cxcl10*, is observed during infectious and inflammatory diseases, playing a crucial role in T-cell-mediated inflammation in the CNS [48]. In addition, *Cxcl10* has a well-established role in inflammatory demyelinating diseases, such as MS, through the destruction of the myelin sheath or neurons by facilitating leukocyte trafficking in the brain [49]. These effects are in agreement with reports showing that JQ1 can modulate the functional activities of immune cells and exert immunosuppressive effects by inhibiting cytokine and chemokine production.

We found that JQ1 significantly suppressed the expression of key LPS-inducible pro-inflammatory enzymes, including *Nos2* and *Ptgs2. Nos2* plays a pivotal role in mediating neuroinflammation to produce NO, a potent proinflammatory mediator, via oxidative deamination [50]. Because neurons and oligodendrocytes are injurious in relation to NO, an oversupply of NO can cause nerve injury in CNS diseases [51]. Thus, drugs that inhibit *Nos2* expression may be possible therapeutic agents for diseases associated with an overproduction NO, including septic shock, inflammation, and neurodegenerative diseases [51]. *Ptgs2* is the key enzyme responsible for brain inflammation, and increased *Ptgs2* expression is believed to contribute to neurodegeneration [52]. In addition, *Ptgs2* is also responsible for the synthesis of inflammation-related PG, and it is believed that the inhibition of PG and NO production might be a therapeutic target for inflammatory diseases such as PD, Huntington’s disease, and AD [53]. In the present study, JQ1 inhibited both *Ptgs2* and *Nos2* expression, and these inhibitory effects of JQ1 may play a potential role in the treatment of neurodegenerative diseases, possibly through its inhibition of microglia and the ensuing inflammatory responses in the CNS. However, the mechanism by which JQ1 inhibits key inflammatory genes requires further study. Interestingly, it has been demonstrated that BET proteins, for example, Brd2 is essential for proinflammatory cytokine production in macrophages and that Brd2, as well as Brd4, physically associates with promoters of inflammatory cytokine genes in macrophages. JQ1-evacuating Brd4 from specific gene promoter renders anti-inflammatory and anti-osteoclastogenetic effects, without ruling out that some effects of JQ1 are derived from inhibiting other members of the BET family [10]. Nevertheless, further investigations are needed to determine selective roles of each BET proteins (Brd2, Brd3, Brd2, and Brdt) through their ability to regulate inflammatory genes.

Another hallmark of inflammation is the increased expression of transcription factors (TFs), such as *Irf1*, *Irf7*, and *Irf9*. Here, we show that JQ1 downregulates the expression of LPS-inducible TFs. Interferon regulatory factors (IRFs) are a family of transcription factors involved in neurological diseases. Type 1 IRFs have well-established roles in neuroinflammation. Indeed, Irf1 and Irf7 are important regulatory factors in the development of demyelination diseases of the CNS, such as MS and experimental autoimmune encephalomyelitis (EAE) [54,55], whereas *Irf9* and *Irf1* are important in injury-induced type 1 IRF signaling, which regulates inflammatory responses in the CNS [56]. Furthermore, JQ1 also inhibited the expression of a wide group of other interferon-stimulated genes (*ISGs*), for example, *Isg15*, *Oasl1*, *Oasl2*, *Ifit-1*, *Ifit-2*, and *Rsad2*, in LPS-stimulated BV-2 microglial cells. Previously, Nicodeme *et al*. [7] reported that another BET protein inhibitor, I-BET, suppressed the expression of *Irf4* and *Irf8* but not *Irf1*, *Irf7*, and *Irf9* in bone marrow-derived macrophages. In the present study, we showed that JQ1 downregulates the expression of *Irf1*, *Irf7*, and *Irf9* and their target genes in BV-2 microglial cells. Thus, the downregulation of *Irf1*, *Irf7*, and *Irf9* through JQ1 could inhibit neurodegenerative diseases as well as brain inflammation. Finally, the results achieved by the RT-PCR analysis of *Irf9*, *Irf1*, *Irak3*, *Ccl2*, *Ccl7*, *Ccl4*, *Ccl12*, *Cxcl10*, *Ptgs2*, *Irg1*, and *Il1a* (Figure 7A,B) illustrate an essential downregulation in the expression of the abovementioned mRNAs in JQ1-treated BV-2 microglial cells when compared to the control. Furthermore, JQ1 downregulates the abovementioned genes in primary microglial cells, under inflammatory conditions (LPS 10 ng/mL) (Figure 8A,B).

In the absence of LPS stimulation, the treatment of BV-2 microglial cells with JQ1 had a marginal effect on gene transcription and did not have an impact on the expression of inflammatory genes (Figure 5). Thus, the impact of JQ1 on LPS-inducible gene expression is highly selective. Most interestingly, crucial inflammatory genes, *Tnf-α* and *Nfκbia* (Additional file 4: Figure S3), as well as other inflammation and immunity-related genes, such as *Saa3*, *Nfkbiz*, *Tnfaip2*, *Nfκb2*, and *Ccl3*, were unaffected by JQ1. This specificity and anti-inflammatory potential of JQ1 was validated by the q-RT-PCR analysis. In our study, we observed prominent transcription factors *Irf1*, *Irf7*, and *Irf9* were suppressed by JQ1, although surprisingly, JQ1 had no effect on master transcription factors *NF-κB* or *AP-1*. Therefore, it seems likely that the LPS-induced induction of *Tnf-α*, *Tnfaip2*, and *Ccl3* transcription depends on *NF-κB* or *AP-1* rather than *Irf1*, *Irf7*, and *Irf9* transcriptional pathways [57-59]. This is an exciting area that we are keenly pursuing further.

Overall, the genome-wide analysis by RNA-Seq identified LPS-inducible genes that were significantly suppressed or unaffected by JQ1, providing a clue for the selective effect of JQ1 on gene expression. However, further extensive in *vivo* experimentation study is required to investigate the anti-inflammatory effect JQ1 and the mechanism by which JQ1 inhibits key inflammatory genes, which will ultimately result in the development of effective and safe anti-inflammatory drugs.

## Conclusion

In summary, this study focused on the anti-inflammatory potential of the synthetic compound JQ1. Our RNA-Seq data for the first time revealed the gene expression profiling of JQ1 in an inflammatory cell model, BV-2 microglia, and targeting inflammatory diseases of the CNS. The findings suggested that JQ1 selectively inhibits the expression of several immune- and inflammation-related genes, including chemokines, interleukins, and interferons, to exert its anti-inflammatory function and that JQ1 could be a candidate for the prevention of inflammation-mediated neurodegenerative diseases.

## Additional files

Additional file 1: Figure S1. Quantification of CD11b positive microglial cells**.** Microglial identification is accomplished using flow cytometry. As quantified by CD11b, 96.27% of cells obtained were microglia. The labeled cells are represented by the pink-shaded populations. Additional file 2:
**Top 25 significant downregulated genes in 2 and 4 h LPS-stimulated BV-2 microglial cells.** **Table S1.** Top 25 significant downregulated genes in 2 h LPS-stimulated BV-2 microglial cells. **Table S2.** Top 25 significant down-regulated genes in 4 h LPS stimulated BV-2 microglial cells. Additional file 3: Figure S2. The inactive enantiomer JQ1 (−) did not reduce cytokine gene expression in BV-2 microglial cells**.** BV-2 microglial cells were exposed simultaneously to JQ1 (−), LPS, and LPS plus JQ1 (−) for 2 and 4 h. Mean value and SEM for the three determinations are shown. Additional file 4: Figure S3. JQ1 did not affect a specific subset of LPS-inducible genes. UCSC Browser images representing the normalized RNA-Seq read density in inflammatory genes un-affected by JQ1 after 2 and 4 h in LPS-stimulated BV-2 microglial cells compared to the control. Additional file 5:
**Top 15 significant upregulated genes in 2 and 4 h JQ1 stimulated BV-2 microglial cells.** **Table S3.** Top 15 significant upregulated genes in 2 h JQ1 stimulated BV-2 microglial cells. **Table S4.** Top 15 significant upregulated genes in 4 h JQ1 stimulated BV-2 microglial cells. Additional file 6: Figure S4. Functional annotation of JQ1-inducible genes. (A and B) Gene Ontology analysis of functional annotations (biological process) associated with 2- and 4-h JQ1-inducible upregulated genes in BV-2 microglial cells in comparison with the control, respectively.
