# Supplementary figures and images for: RNA sequencing reveals distinct mechanisms underlying BET inhibitor JQ1-mediated modulation of the LPS-induced activation of BV-2 microglial cells
Source: J Neuroinflammation. 2015 Feb 24;12:36. doi: 10.1186/s12974-015-0260-5 (PMC4359438; doi:10.1186/s12974-015-0260-5)

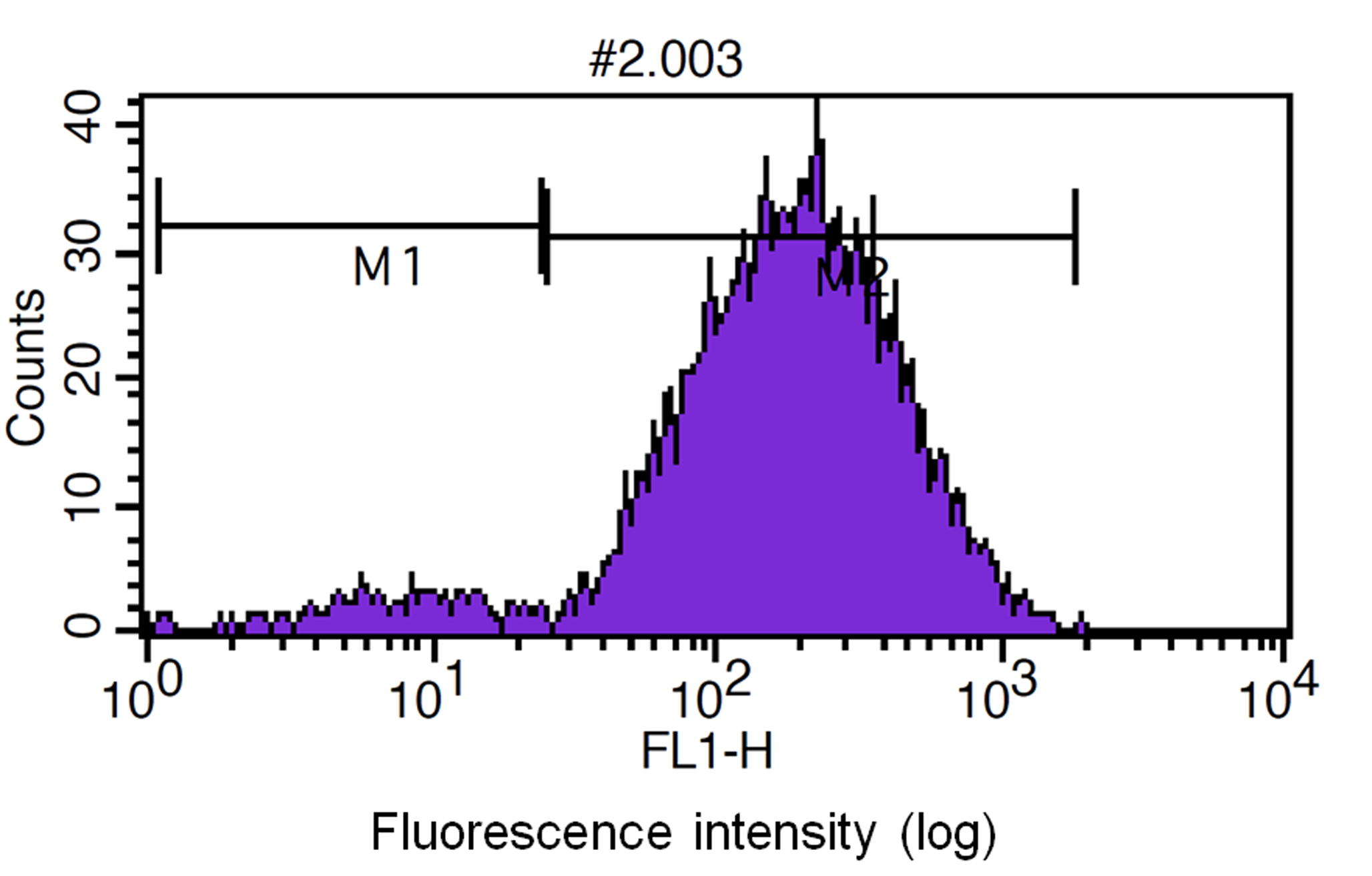

Supplement: Additional file 1: Figure S1. — Quantification of CD11b positive microglial cells. Microglial identification is accomplished using flow cytometry. As quantified by CD11b, 96.27% of cells obtained were microglia. The labeled cells are represented by the pink-shaded populations. [file 12974_2015_260_MOESM1_ESM.tiff]

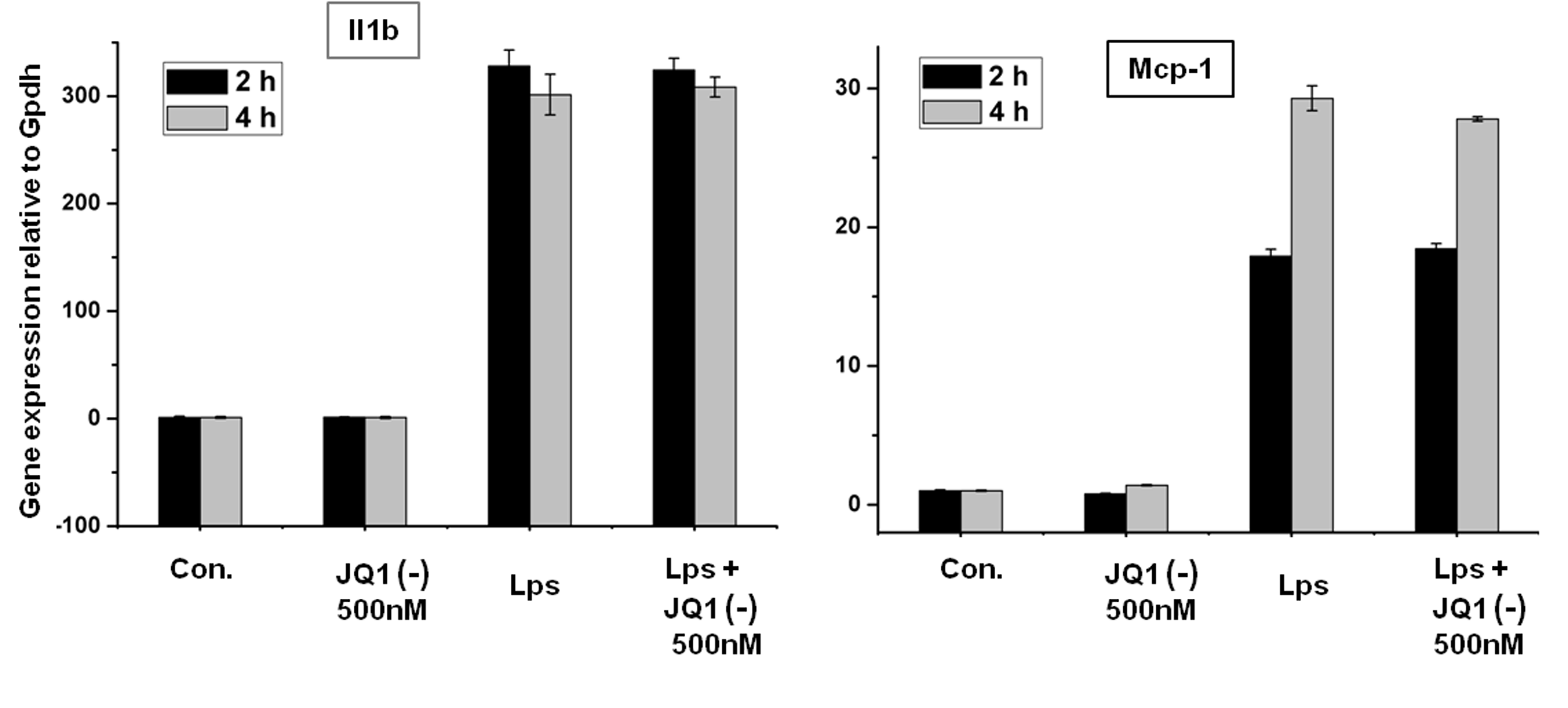

Supplement: Additional file 3: Figure S2. — The inactive enantiomer JQ1 (−) did not reduce cytokine gene expression in BV-2 microglial cells. BV-2 microglial cells were exposed simultaneously to JQ1 (−), LPS, and LPS plus JQ1 (−) for 2 and 4 h. Mean value and SEM for the three determinations are shown. [file 12974_2015_260_MOESM3_ESM.tiff]

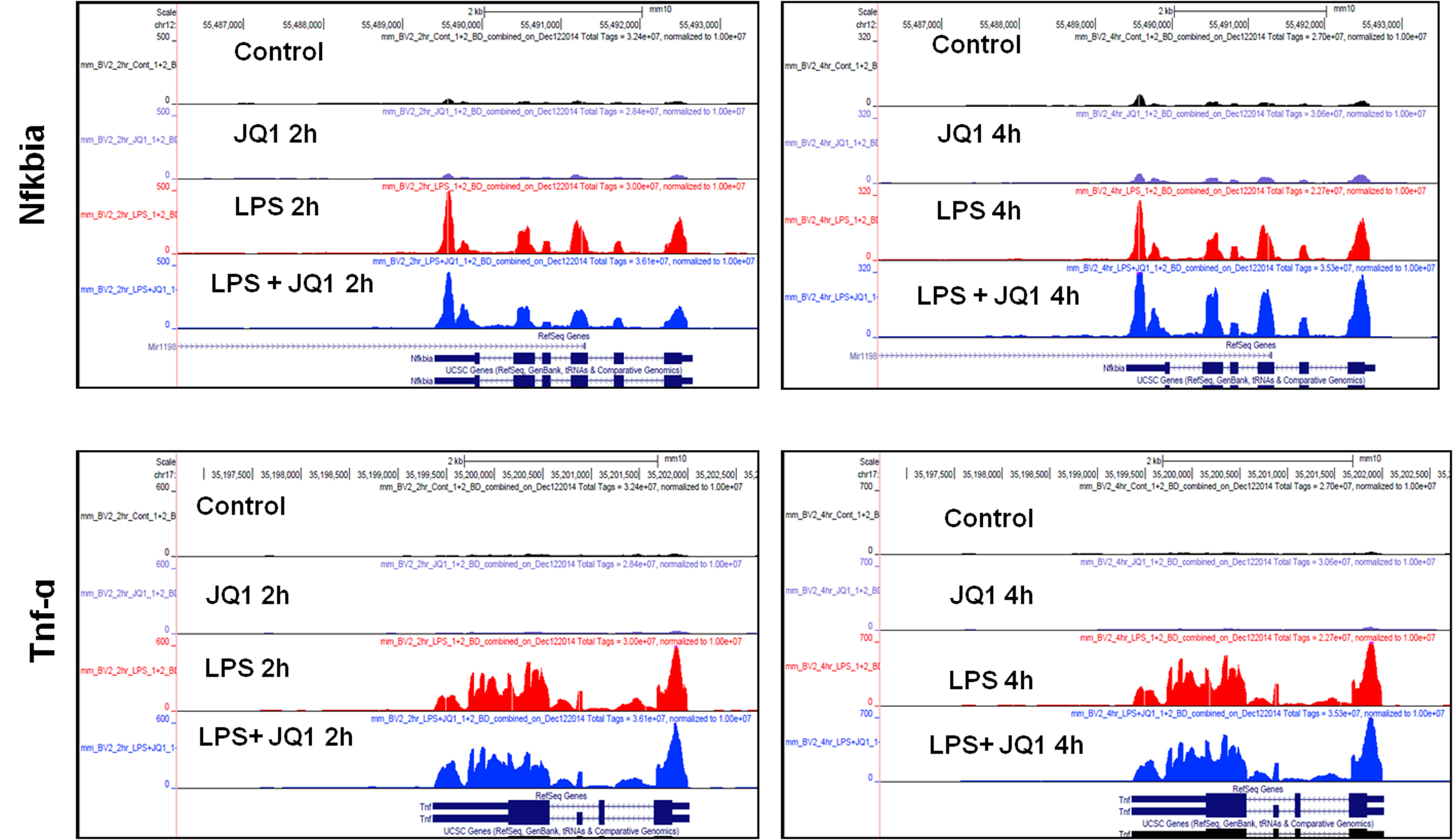

Supplement: Additional file 4: Figure S3. — JQ1 did not affect a specific subset of LPS-inducible genes. UCSC Browser images representing the normalized RNA-Seq read density in inflammatory genes un-affected by JQ1 after 2 and 4 h in LPS-stimulated BV-2 microglial cells compared to the control. [file 12974_2015_260_MOESM4_ESM.tiff]

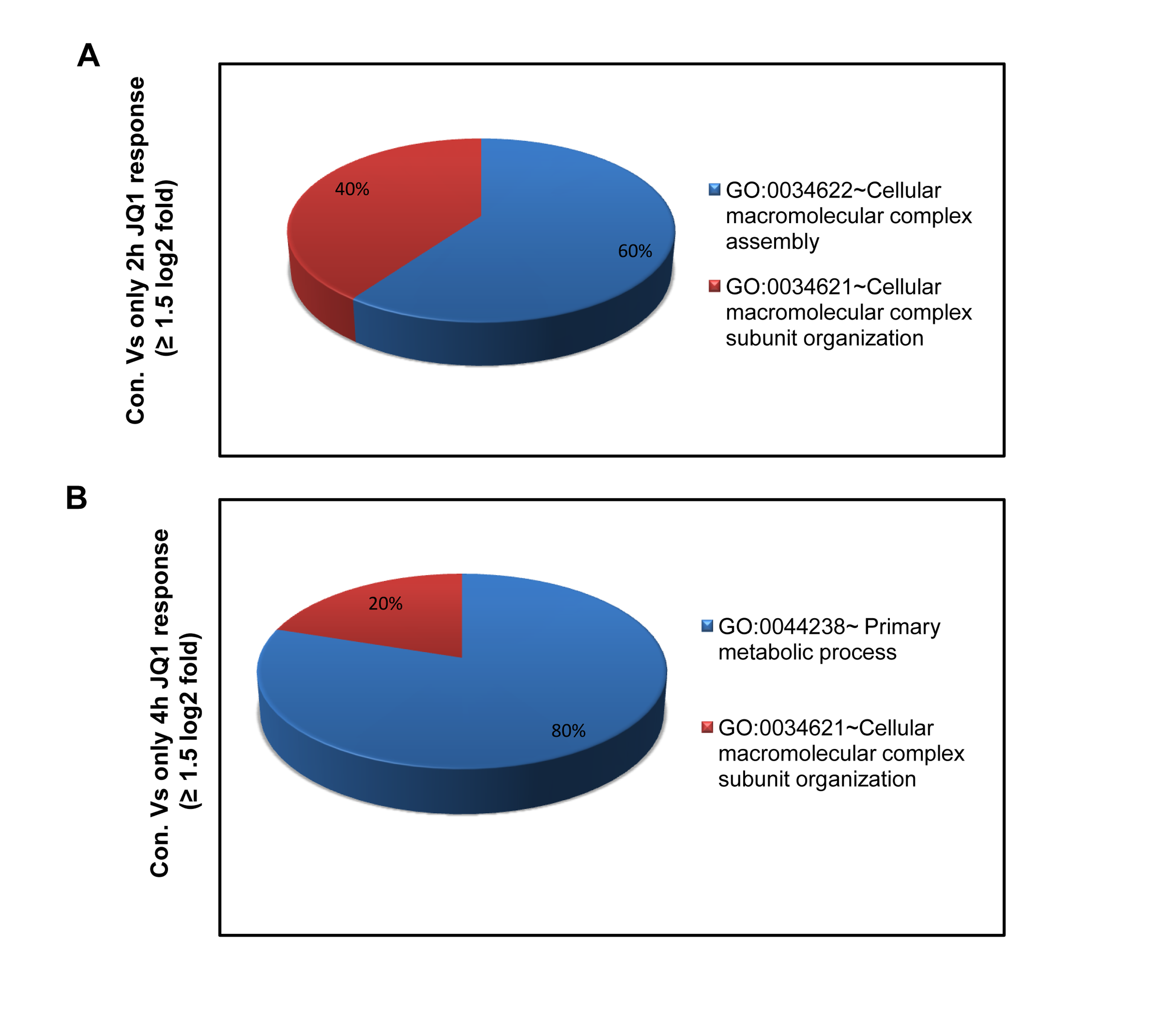

Supplement: Additional file 6: Figure S4. — Functional annotation of JQ1-inducible genes. (A and B) Gene Ontology analysis of functional annotations (biological process) associated with 2- and 4-h JQ1-inducible upregulated genes in BV-2 microglial cells in comparison with the control, respectively. [file 12974_2015_260_MOESM6_ESM.tiff]
