# Supplementary material for: RNA sequencing reveals distinct mechanisms underlying BET inhibitor JQ1-mediated modulation of the LPS-induced activation of BV-2 microglial cells
Source: J Neuroinflammation. 2015 Feb 24;12:36. doi: 10.1186/s12974-015-0260-5 (PMC4359438; doi:10.1186/s12974-015-0260-5)
Supplement: Additional file 2: — Top 25 significant downregulated genes in 2 and 4 h LPS-stimulated BV-2 microglial cells. Table S1. Top 25 significant downregulated genes in 2 h LPS-stimulated BV-2 microglial cells. Table S2. Top 25 significant down-regulated genes in 4 h LPS stimulated BV-2 microglial cells. [file 12974_2015_260_MOESM2_ESM.doc]

**Table S1:** Top 25 significant down-regulated genes in 2 h LPS stimulated BV-2 microglial cells

| **Gene Accession_ID** | **Gene Symbol** | **Expression**  **log2 (fold_change)** | **P-Value** |
| --- | --- | --- | --- |
| NM_183031 | Gpr183 | -4.66695 | 0.0001 |
| NM_001113470 | Ctdsp2 | -4.31035 | 0.0001 |
| NM_009378 | Thbd | -3.56323 | 0.00195 |
| NM_175542 | Rttn | -3.52428 | 0.00005 |
| NM_001177973 | Irak1 | -3.31097 | 0.00005 |
| NM_009061 | Rgs2 | -3.27504 | 0.00005 |
| NM_026268 | Dusp6 | -3.25757 | 0.0002 |
| NM_009911 | Cxcr4 | -2.89439 | 0.00005 |
| NM_001038703 | Gpr146 | -2.6869 | 0.00191 |
| NM_199221 | Cd300lb | -2.61939 | 0.00005 |
| NM_175116 | Lpar6 | -2.59604 | 0.00785 |
| NM_001168615 | Tifab | -2.58859 | 0.00005 |
| NM_001271768 | Bhlhe41 | -2.47596 | 0.0001 |
| NM_029116 | Kbtbd11 | -2.47361 | 0.00045 |
| NM_028724 | Rin2 | -2.46475 | 0.00005 |
| NM_001039106 | Ddhd1 | -2.44762 | 0.00013 |
| NM_133687 | Cxxc5 | -2.42205 | 0.0002 |
| NM_013926 | Cbx8 | -2.37976 | 0.0001 |
| NM_010658 | Mafb | -2.28992 | 0.00015 |
| NM_199029 | Zfp395 | -2.25189 | 0.0009 |
| NM_021325 | Cd200r1 | -2.20084 | 0.000125 |
| NM_177337 | Arl11 | -2.19169 | 0.00075 |
| NM_001146005 | C5ar2 | -2.18178 | 0.00055 |
| NM_001114679 | 9930111J21Rik1 | -2.15836 | 0.00035 |
| NM_009924 | Cnr2 | -2.14268 | 0.00055 |

**Table S2:** Top 25 significant down-regulated genes in 4 h LPS stimulated BV-2 microglial cells

| **Gene Accession_ID** | **Gene Symbol** | **Expression**  **log2 (fold_change)** | **P-Value** |
| --- | --- | --- | --- |
| NM_009911 | Cxcr4 | -5.19885 | 0.0001 |
| NM_029116 | Kbtbd11 | -4.7056 | 0.0011 |
| NM_029999 | Lbh | -3.6828 | 0.00025 |
| NM_001130412 | Lpin1 | -3.49501 | 0.00005 |
| NM_177708 | Rtn4rl1 | -3.49324 | 0.000365 |
| NM_013867 | Bcar3 | -3.38281 | 0.00005 |
| NM_026268 | Dusp6 | -3.25757 | 0.001 |
| NM_080444 | Asb10 | -3.38185 | 0.0002 |
| NM_011994 | Abcd2 | -3.26676 | 0.00005 |
| NM_199029 | Zfp395 | -3.25155 | 0.0003 |
| NM_026185 | Abhd15 | -3.1729 | 0.00095 |
| NM_139200 | Cytip | -3.15384 | 0.00005 |
| NM_138313 | Bmf | -3.15159 | 0.00005 |
| NM_001039106 | Ddhd1 | -3.11967 | 0.0001 |
| NM_008587 | Mertk | -3.07958 | 0.0012 |
| NM_133687 | Cxxc5 | -3.07476 | 0.00915 |
| NM_001290148 | Snx29 | -3.06313 | 0.00005 |
| NM_177366 | Gpr157 | -3.05206 | 0.00005 |
| NM_001271386 | Hdac9 | -3.00687 | 0.00005 |
| NM_183088 | Zbed5 | -2.98923 | 0.0015 |
| NM_183031 | Gpr183 | -2.98705 | 0.0011 |
| NM_133212 | Tlr8 | -2.9781 | 0.0002 |
| NM_183390 | Klhl6 | -2.94205 | 0.0011 |
| NM_130860 | Cdk9 | -2.93467 | 0.00005 |
| NM_009645 | Aicda | -2.90497 | 0.0008 |
