# Supplementary material for: RNA sequencing reveals distinct mechanisms underlying BET inhibitor JQ1-mediated modulation of the LPS-induced activation of BV-2 microglial cells
Source: J Neuroinflammation. 2015 Feb 24;12:36. doi: 10.1186/s12974-015-0260-5 (PMC4359438; doi:10.1186/s12974-015-0260-5)
Supplement: Additional file 5: — Top 15 significant upregulated genes in 2 and 4 h JQ1 stimulated BV-2 microglial cells. Table S3. Top 15 significant upregulated genes in 2 h JQ1 stimulated BV-2 microglial cells. Table S4. Top 15 significant upregulated genes in 4 h JQ1 stimulated BV-2 microglial cells. [file 12974_2015_260_MOESM5_ESM.doc]

**Table S3:** Top 15 significant up-regulated genes in 2 h JQ1 stimulated BV-2 microglial cells

| **Gene Accession_ID** | **Gene Symbol** | **Expression**  **log2 (fold_change)** | **P-Value** |
| --- | --- | --- | --- |
| [NM_001039106](http://www.ncbi.nlm.nih.gov/nuccore/NM_001039106.3) | Ddhd1 | 3.84241 | 0.006 |
| [NM_001286404](http://www.ncbi.nlm.nih.gov/nuccore/NM_001286404.1) | Ghrl | 3.63639 | 0.019 |
| [NR_037682](http://www.ncbi.nlm.nih.gov/nuccore/NR_037682.1) | Snord42a | 2.97813 | 0.002 |
| [NM_001195258](http://www.ncbi.nlm.nih.gov/nuccore/NM_001195258.1) | Gm14378 | 2.61777 | 0.002 |
| [NR_045266](http://www.ncbi.nlm.nih.gov/nuccore/NR_045266.1) | Gm15545 | 2.51945 | 0.005 |
| [NM_170591](http://www.ncbi.nlm.nih.gov/nuccore/NM_170591.1) | Nupl1 | 2.3784 | 0.001 |
| [NR_028532](http://www.ncbi.nlm.nih.gov/nuccore/NR_028532.1) | Snord71 | 2.26305 | 0.005 |
| [NM_178337](http://www.ncbi.nlm.nih.gov/nuccore/NM_178337.2) | Tbce | 2.2416 | 0.001 |
| [NM_207541](http://www.ncbi.nlm.nih.gov/nuccore/NM_207541.1) | Zfp81 | 2.19193 | 0.016 |
| [NR_028129](http://www.ncbi.nlm.nih.gov/nuccore/NR_028129.1) | AF357399 | 2.1526 | 0.001 |
| [NM_144816](http://www.ncbi.nlm.nih.gov/nuccore/NM_144816.1) | Rhbdl1 | 2.04137 | 0.0042 |
| [NM_001113470](http://www.ncbi.nlm.nih.gov/nuccore/NM_001113470.1) | Ctdsp2 | 2.00976 | 0.001 |
| [NR_033215](http://www.ncbi.nlm.nih.gov/nuccore/NR_033215.1) | 3000002C10Rik | 2.00233 | 0.003 |
| [NM_001033419](http://www.ncbi.nlm.nih.gov/nuccore/NM_001033419.2) | Ceacam16 | 1.92161 | 0.001 |
| [NM_029431](http://www.ncbi.nlm.nih.gov/nuccore/NM_029431.1) | Them4 | 1.91483 | 0.002 |

**Table S4:** Top 15 significant up-regulated genes in 4 h JQ1 stimulated BV-2 microglial cells

| **Gene Accession_ID** | **Gene Symbol** | **Expression**  **log2 (fold_change)** | **P-Value** |
| --- | --- | --- | --- |
| [NM_001163478](http://www.ncbi.nlm.nih.gov/nuccore/NM_001163478.1) | Rabggtb | 3.7333 | 0.002 |
| [NM_001005331](http://www.ncbi.nlm.nih.gov/nuccore/NM_001005331.1) | Eif4g1 | 3.57181 | 0.003 |
| [NR_028558](http://www.ncbi.nlm.nih.gov/nuccore/NR_028558.1) | Snora41 | 3.20642 | 0.004 |
| [NR_028545](http://www.ncbi.nlm.nih.gov/nuccore/NR_028545.1) | Scarna8 | 3.07356 | 0.001 |
| [NM_001123037](http://www.ncbi.nlm.nih.gov/nuccore/NM_001123037.2) | Eif4a2 | 2.91675 | 0.005 |
| [NM_009101](http://www.ncbi.nlm.nih.gov/nuccore/NM_009101.2) | Rras | 2.58505 | 0.005 |
| [NR_028518](http://www.ncbi.nlm.nih.gov/nuccore/NR_028518.1) | Scarna3a | 2.52629 | 0.002 |
| [NM_001290701](http://www.ncbi.nlm.nih.gov/nuccore/NM_001290701.1) | Meaf6 | 2.40336 | 0.007 |
| [NM_001039122](http://www.ncbi.nlm.nih.gov/nuccore/NM_001039122.1) | Defb25 | 2.32067 | 0.006 |
| [NM_033146](http://www.ncbi.nlm.nih.gov/nuccore/NM_033146.1) | Emc9 | 2.24519 | 0.002 |
| [NM_199308](http://www.ncbi.nlm.nih.gov/nuccore/NM_199308.2) | Mast3 | 2.22452 | 0.01 |
| [NM_001146022](http://www.ncbi.nlm.nih.gov/nuccore/NM_001146022.2) | Wdfy4 | 2.15439 | 0.017 |
| [NM_029508](http://www.ncbi.nlm.nih.gov/nuccore/NM_029508.3) | Pcgf5 | 2.07597 | 0.013 |
| [NM_026536](http://www.ncbi.nlm.nih.gov/nuccore/NM_026536.1) | Atp5s | 2.03062 | 0.002 |
| [NR_015464](http://www.ncbi.nlm.nih.gov/nuccore/NR_015464.1) | A330069E16Rik | 2.01805 | 0.014 |
